# Artificial Intelligence (AI) for Programmed Death Ligand-1 (PD-L1) Immunohistochemical Assessment in Urothelial Carcinomas: “Teaching” Cell Differentiation to AI Systems

**DOI:** 10.3390/life15060839

**Published:** 2025-05-22

**Authors:** Ioan Alin Nechifor-Boilă, Adela Nechifor-Boilă, Andrada Loghin, Carmen Mihaela Mihu, Carmen Stanca Melincovici, Mădălin Mihai Onofrei, Călin Bogdan Chibelean, Orsolya Martha, Angela Borda

**Affiliations:** 1 Department of Anatomy and Embryology, George Emil Palade University of Medicine, Pharmacy, Science and Technology of Targu-Mures, 38th Gh. Marinescu Street, 540142 Targu-Mures, Romania; ioan.nechifor-boila@umfst.ro; 2 Department of Urology, Targu-Mures County Hospital, 1st Gh. Marinescu Street, 540103 Targu-Mures, Romania; calin.chibelean@umfst.ro (C.B.C.); orsolya.martha@umfst.ro (O.M.); 3 Department of Histology, Center for Advanced Medical and Pharmaceutical Research, George Emil Palade University of Medicine, Pharmacy, Science and Technology of Targu-Mures, 38th Gh. Marinescu Street, 540142 Targu-Mures, Romania; andrada.loghin@umfst.ro (A.L.); angela.borda@umfst.ro (A.B.); 4 Department of Pathology, Targu-Mures County Hospital, 28 1st December 1918 Boulevard, 540011 Targu-Mures, Romania; 5 Discipline of Histology, Department of Morpho-Functional Sciences, Iuliu Hatieganu University of Medicine and Pharmacy, 400349 Cluj-Napoca, Romania; carmenmihu@umfcluj.ro (C.M.M.); carmen.melincovici@umfcluj.ro (C.S.M.); madalin.onofrei@umfcluj.ro (M.M.O.); 6 Department of Urology, George Emil Palade University of Medicine, Pharmacy, Science and Technology of Targu-Mures, 38th Gh. Marinescu Street, 540142 Targu-Mures, Romania; 7 Department of Pathology, Targu-Mures Emergency County Hospital, 50th Gh. Marinescu Street, 540136 Targu-Mures, Romania

**Keywords:** PD-L1, bladder cancer, immunohistochemistry, artificial intelligence, QuPath, whole slide imaging

## Abstract

Assessment of Programmed Death-Ligand 1 (PD-L1) immunohistochemical (IHC) expression on tumor cells (TCs) and immune cells (ICs) in bladder cancer (BC) is challenging. Artificial Intelligence (AI) has potential for accurate PD-L1 IHC scoring, but its efficiency remains debatable. Our aim was to compare two AI protocols provided by the free QuPath software (v0.5.1) (Selected Area Interpretation (AI-SAI) and Whole Slide Imaging (AI-WSI)) with manual PD-L1 IHC scoring. A total of 43 BCs were included. PD-L1 IHC was performed using the SP263 clone. The IHC slides were digitized and further imported into QuPath. The PD-L1 positivity threshold was set at 25%. Statistically significant correlations were observed between AI-SAI and manual interpretation for both TCs (r = 0.85) and ICs (r = 0.57). AI-WSI yielded comparable results, with correlation coefficients of r = 0.82 for TCs and r = 0.56 for ICs. However, AI-SAI demonstrated stronger agreement with manual assessment (κ = 0.86) compared to AI-WSI (κ = 0.65). Receiver Operating Characteristic (ROC) analysis further supported the superiority of AI-SAI, with higher AUC values for both TCs (0.96 vs. 0.92) and ICs (0.92 vs. 0.90). Our findings indicate that AI-SAI is preferable to AI-WSI, particularly in BC cases with high PD-L1-positive TC content. Nevertheless, supervision by an experienced pathologist is mandatory.

## 1. Introduction

In recent years, immunotherapy by checkpoint blockade targeting programmed cell death protein-1 [PD-1] or its ligand (programmed death ligand-1 [PD-L1]) has reshaped the treatment of advanced cancer around the world, showing remarkable clinical activity in several tumor types [1]. In the case of urothelial carcinoma (UC), namely bladder cancer (BC), immunotherapy is currently recommended as a second-line treatment for patients with unresectable or metastatic tumors. Additionally, it also serves as a first-line option for patients who are ineligible for cisplatin-based chemotherapy, the standard first-line treatment [2,3]. Moreover, recent data have revealed promising results for immunotherapy both as a neoadjuvant and adjuvant treatment in BC cases. However, according to Food and Drug Administration (FDA) and European Medicines Agency (EMA) guidelines, the use of PD-1/PD-L1 inhibitors requires a positive PD-L1 status assessed by immunohistochemistry (IHC) [4,5,6].

Currently, there are four validated, commercially available IHC assays for PD-L1 IHC evaluation: 22C3 and 28-8 from Dako (Agilent, St. Clara, CA, USA), and SP142 and SP263 from Ventana Medical Systems (Oro Valley, AZ, USA), each with its own staining protocol and scoring algorithm. The PD-L1 IHC SP263 clone is used in conjunction with the drug Durvalumab (Imfizi™, AstraZeneca, Södertälje, Sweden), as a companion diagnostic assay. Its current interpretation system relies on the assessment of the percentage of PD-L1-positive tumor cells (TCs) and/or tumor-infiltrating immune cells (ICs) using 25% as the cut-off value [7].

It is recognized that PD-L1 IHC manual interpretation can be difficult, and literature data indicate significant intra- and interobserver variability, which is heavily reliant on the pathologist’s expertise [1,8], a factor that could influence the predictive value of PD-L1 testing.

Automatic scoring using digital slide images has the potential to provide high-throughput PD-L1 IHC scoring with promising results in non-small cell lung cancer (NSCLC) and BC, as indicated by recent literature data [9,10]. The performance of these tests may vary based on the software and hardware available in different laboratories. Previous studies utilizing privately owned software or in-house-developed scoring algorithms have shown that PD-L1 IHC scores can be comparable to those obtained through manual interpretation. However, the practical application of this software in routine practice remains inconsistent, largely due to funding considerations, which play a significant role in selecting a software suite [9,11]. Community-driven, open-source software such as QuPath (v0.5.1) may provide a more accessible and cost-effective alternative for automated PD-L1 immunohistochemistry (IHC) scoring. However, the current body of research investigating the application of QuPath in this context remains limited, particularly in BC, with most studies to date focusing on NSCLC. Despite this, preliminary findings are encouraging, indicating that automated PD-L1 IHC assessment using QuPath demonstrates performance comparable to manual evaluation by experienced pathologists [10]. These studies have primarily concentrated on PD-L1 IHC interpretation using tissue microarrays or small tissue regions, known as Selected Area Interpretation (SAI). However, this approach may be inadequate. It underscores the necessity of evaluating the entire tissue on the slide through whole slide imaging (WSI) [11].

The aim of the current study was to evaluate the efficiency of PD-L1 IHC interpretation using AI tools available in the free open-source QuPath software. This evaluation included both the AI-SAI and AI-WSI protocols, and the results were compared to a manual scoring evaluation, which served as the gold standard.

## 2. Materials and Methods

### 2.1. Case Selection

This study was approved by the Ethics Committee of the George Emil Palade University of Medicine, Pharmacy, Science, and Technology of Targu Mures, Romania (letter of decision no. 2969/25 March 2024).

All patients with BC that underwent radical cystectomy in the Department of Urology, Targu Mures County Hospital, Romania, between November 2011 and October 2018, were re-evaluated for inclusion in the study. Inclusion criteria were as follows: (1) a histopathological diagnosis consistent with BC; (2) a tumor stage of at least pT2; (3) no neoadjuvant chemotherapy or radiotherapy received prior to radical cystectomy; (4) availability of hematoxylin/eosin (HE) slides for case review; and (5) well-preserved formalin-fixed and paraffin-embedded (FFPE) tumor blocks in the archives for IHC assay.

The methodology followed protocols from our previous studies, including initial HE reviews and IHC staining procedures [12,13].

Following the application of inclusion and exclusion criteria, a total of 43 patients were deemed eligible and included in the study.

The corresponding HE slides for all the cases included in the study were reviewed by three expert uropathologists (A.N.-B., A.L., and A.B.) on a three-headed microscope (Leica DM2500, Leica Microsystems GmbH, Wetzlar, Germany) in consensus. Tumor histology, grade, and TNM staging were reassessed using the 2016 WHO (World Health Organization) Classification of Tumors of the Urinary System and Male Genital Organs [14] as well as the 2017 American Joint Committee on Cancer/Union for International Cancer Control (AJCC/UICC) TNM classification of tumors [15]. For each case, one representative FFPE block with clear areas of tumor was selected for the IHC assay. The tumor block corresponded to well-preserved tumor areas, with at least 100 viable TCs in the sample and the absence of hemorrhage and/or necrosis.

### 2.2. IHC Assay for Detection of PD-L1 Expression

IHC was performed on 4 μm thick full sections. Staining was performed on a Benchmark Ultra System (Ventana (Oro Valley, AZ, USA), Roche (Basel, Switzerland)) with antibody visualization using the Optic View DAB (diaminobenzidine) IHC Detection Kit (Ventana), according to the manufacturer’s instructions and adjusted in our lab. The following were applied for the SP263 clone (ready to use; rabbit, monoclonal, Ventana, Roche): Heat-Induced Epitope Retrieval at 100 °C in EDTA (pH = 9) buffer for 64 min, and primary antibody incubation for 16 min at 37 °C.

Positive staining was defined as partial or complete membranous staining at any intensity for tumor cells (TCs) and cytoplasmic or membranous staining of any intensity for immune cells (ICs). Positive controls (tonsil tissue) were included in every run.

### 2.3. Manual Scoring Assessment of PD-L1 Expression

PD-L1 manual scoring assessment was performed by three pathologists with a special interest in uropathology (A.N.-B., A.L., and A.B.). To reduce potential interobserver variability in the assessment process, all cases were evaluated in a panel, and a consensus was reached when examining each case.

The entire surface of the IHC slide was evaluated, and the percentage of positive TCs and ICs was set by calculating the percentage of PD-L1 immunoreactive cells (TCs and ICs, including leukocytes and macrophages) within the entire tumor area but more precisely in the invasion front. As recommended by the Ventana PD-L1 (SP263) Assay in Urothelial Carcinoma Interpretation Guide [7], the PD-L1 IHC positivity cut-off value was set at ≥25% for positive TCs and/or ICs.

### 2.4. Slide Scanning and Automatic Scoring Assessment of PD-L1 Expression

A Leica Biosystems Aperio LV1 instrument (Leica, Wetzlar, Germany; Department of Histology, Iuliu Hațieganu University of Medicine and Pharmacy in Cluj-Napoca) was used to scan the IHC slides at 40× magnification. Each one was then imported separately into QuPath software. The interpretation was carried out through the following steps:

(1) Training the software classifier by means of selected representative areas for both TCs and ICs that were identified and marked on the scanned slide by a pathologist (A.N.-B.);
(2) Case interpretation and PD-L1 scoring assessment using Selected Area Interpretation (SAI) only (AI-SAI protocol);
(3) Re-interpretation of the case and new PD-L1 scoring assessment including the entire tissue area present on the slide, using Whole Slide Imaging (WSI; AI-WSI protocol).

For training, the pathologist selected 1 to 3 annotated areas that were considered representative for the case, with the magnification set at 4×. The software subsequently identified cells within the designated annotations. Technically, the stains were separated through color deconvolution, and nuclei were identified based on standard user-defined parameters in the hematoxylin channel. The cell count was approximated by evaluating the proximity of neighboring nuclei and applying specified parameters. A random trees classifier was developed using input from various cellular areas. Consequently, multiple cellular regions were annotated within the detection zones, and the pathologist classified them as either TCs or ICs.

**Figure 1:**
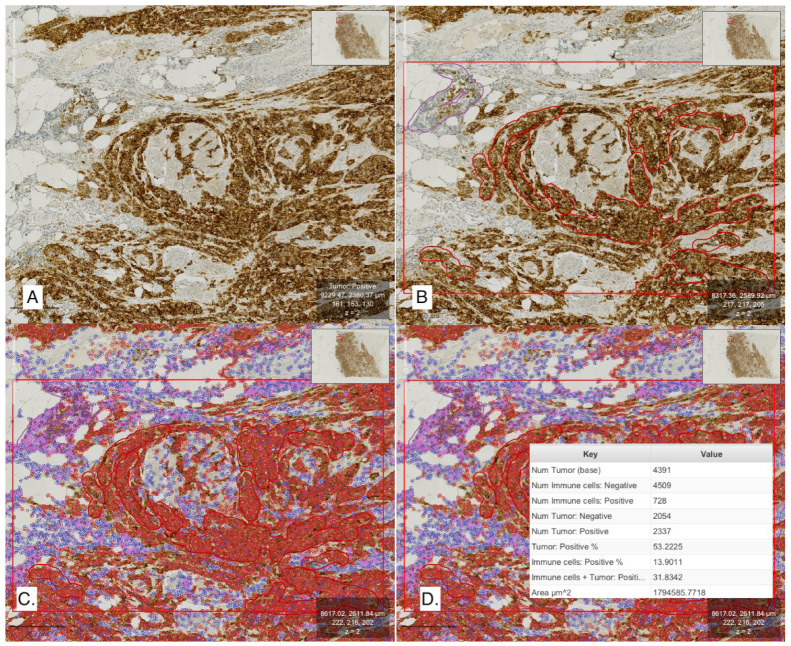
Description of AI-SAI protocol. The automatic interpretation process begins with the training of the classifier. After selecting a representative area on the slide (**A**), a large annotation is drawn to encompass the entire region (AI-SAI). Following the application of the cell detection tool, smaller annotations are added within the larger one to include representative samples of tumor cells (TCs, red) and immune cells (ICs, purple) (**B**). The characteristics of these areas are manually input into the classifier. Subsequently, the positive cell detection command is executed, followed by the classifier (**C**), and the summary of the large annotation displays the percentage of positive TCs and ICs (**D**). AI-SAI: Artificial Intelligence protocol, Selected Area Interpretation; TC: tumor cell; IC: immune cell.

**Figure 2:**
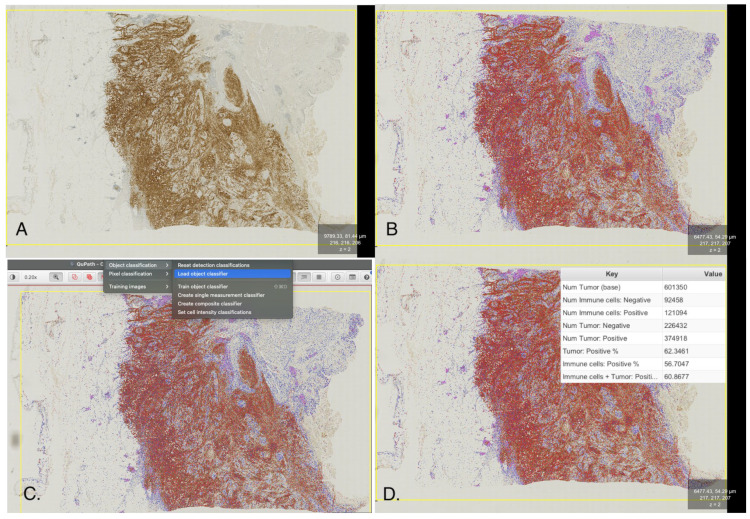
The AI-WSI protocol involves the following steps: First, all tissue on the slide is annotated (**A**). Next, the positive cell detection command is used (**B**). The cells are then classified using the previously trained classifier (**C**), and the final results are summarized (**D**). AI-WSI: Artificial Intelligence, Whole Slide Imaging.

A total of 729 areas were selected, including all 43 study cases with an average of one or two detection zones per case. This information was incorporated into the classifier. The selected zones and the cell detection annotations were graphically visible for optical inspection. Subsequently, the positive cell detection tool was used, and the classifier was applied. Positive cells were determined by their optical DAB (diaminobenzidine) intensity exceeding a predetermined threshold. The percentages of positive TCs and ICs were obtained (Figure 1 and Figure 2).

For SAI interpretation (AI-SAI protocol), cells from the initial detection zones were analyzed using both the positive cell detection tool and the classifier (Figure 1). The percentage of positive TCs and ICs was evaluated, classifying each case as positive or negative based on a 25% positivity cut-off.

Next, a new annotation including all tissue on the slide was created (AI-WSI protocol). The entire process (cell identification, classification, and percentage calculation) was repeated for the whole slide (Figure 2). Each case was re-classified using the 25% positivity cut-off. Thus, each case received three interpretations for SP263 immunohistochemical staining: manual, AI-SAI, and AI-WSI [7].

### 2.5. Statistical Analysis

Data analysis was conducted utilizing Jamovi software version 2.5 for Mac [16], in conjunction with IBM SPSS Statistics version 25 (SPSS Inc., Armonk, NY, USA). To evaluate the consistency between manual and automated methods for assessing positive PD-L1 staining, Spearman correlation coefficients (r) were calculated pairwise for both tumor cells (TCs) and immune cells (ICs) in each case: manual assessment versus AI-SAI, as well as manual assessment versus AI-WSI. The obtained correlation value was defined as follows: (1) *very good* if r > 0.75 or <−0.75; (2) *moderate to good* if r was between 0.75 and 0.5 or between −0.75 and −0.5; (3) *acceptable* if r was between 0.25 and 0.5 or between −0.5 and −0.25; and (4) *low or no correlation* if r was between −0.25 and 0.25.

Cohen’s kappa statistic with the Altman (1991) [17] interpretation system was applied to determine both the concordance between the percentages of positive cells (TCs and ICs) and the final PD-L1 IHC scoring as assessed using automatic (both AI-SAI and AI-WSI protocols) versus manual scoring interpretation.

Sensitivity, specificity, positive likelihood ratios, and negative likelihood ratios were computed for both the AI-SAI and AI-WSI protocols, using manual interpretation as the gold standard. Receiver Operating Characteristic (ROC) curves were generated for direct comparison between the AI-SAI and AI-WSI protocols, and the corresponding Area Under the Curve (AUC) was calculated.

## 3. Results

### 3.1. Characteristics of the Study Population

Forty-three patients with BC that underwent surgery in the Department of Urology, Mures County Hospital, Targu Mures, Romania, between November 2011 and October 2018 were included in our study. The mean age at diagnosis was 63.5 ± 7.4 years old, and most patients were men (*n* = 36, 84%) (Table 1).

In terms of histopathological diagnosis, approximately half of the cases were conventional UCs (*n* = 23, 53.5%), followed by UC variants, with the poorly differentiated (*n* = 6, 14%) and squamous (*n* = 4, 9.3%) ones being the most frequent. Most of the cases were pT3 (*n* = 20, 46.5%), and lymph node involvement was documented in 68.7% (*n* = 30) of the UC patients.

### 3.2. Comparative Analysis of AI Protocols Versus Manual Evaluation of TC and IC Staining

We compared the TC and IC staining results as assessed by AI algorithms to those obtained via manual interpretation, which was used as the gold standard.

In our study, 39.5%, 44.18%, and 32.5% of BCs exhibited positive staining of the TCs as evaluated by the manual interpretation, AI-SAI, and AI-WSI protocols, respectively, with the positivity cut-off set at 25%. The differences were statistically significant (*p* = 0.008) (Table 2). There were also significant differences in the percentages of BCs exhibiting positive staining of ICs (cut-off set at 25%) when assessed with the manual (6.9%) versus automatic AI methods (39.5% and 34.8% for AI-SAI and AI-WSI protocols, respectively) (*p* < 0.001) (Table 2).

By using the paired Spearman method, we found a statistically significant correlation (r = 0.85, *p* < 0.001) for TC evaluation between the AI-SAI protocol and the manual method. For the AI-WSI protocol, our data also showed a statistically significant correlation (r = 0.82, *p* < 0.001) between the percentages of positive TCs for the automated and manual interpretations.

A “moderate to good” correlation (r = 0.57, *p* < 0.001) was observed between AI-SAI and manual interpretation when examining the ICs. A similar “moderate to good” statistically significant correlation (r = 0.56, *p* < 0.001) was found between AI-WSI and the manual method.

**Figure 3:**
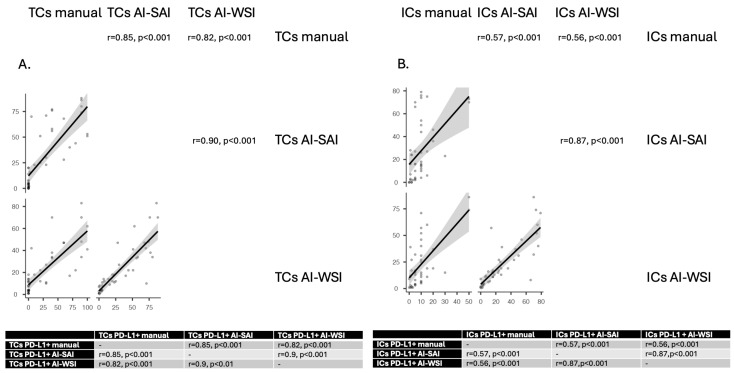
Paired Spearman correlations for assessing tumor (**A**) and immune cells (**B**) with the three scoring methods: manual versus AI-driven protocols (AI-SAI and AI-WSI). The correlations between manual interpretation, AI-SAI, and AI-WSI were explored using the Spearman test. Strong, significant correlations were obtained for TCs in all three interpretation protocols (part (**A**) and table). For ICs, we found moderate to good correlations, all statistically significant (part (**B**) and table). AI: Artificial Intelligence protocol; SAI: Selected Area Interpretation; WSI: Whole Slide Imaging; TCs: tumor cells; ICs: immune cells; PD-L1: Programmed Death Ligand 1.

The results of this comparative analysis are illustrated in Figure 3A,B.

### 3.3. Comparative Analysis of AI Protocols Versus Manual Evaluation in the Assessment of PD-L1 IHC Expression

For both the manual and AI scoring assessments of PD-L1 IHC expression, a 25% positivity cut-off value for TCs and/or ICs was applied. Thus, a case was classified as positive if ≥25% PD-L1-positive TCs and/or ICs were identified in the examined specimen.

After the final case classification, manual interpretation identified 18 (42%) PD-L1-positive and 25 (58%) PD-L1-negative BC cases. According to the AI-SAI protocol, 21 (48.8%) BC cases were classified as PD-L1-positive and 22 (51.2%) as PD-L1-negative. When applying the AI-WSI protocol, 15 (34.9%) BCs were identified as PD-L1-positive and 28 (65.1%) as PD-L1-negative. The differences were statistically significant (*p* = 0.034, Table 2).

**Figure 4:**
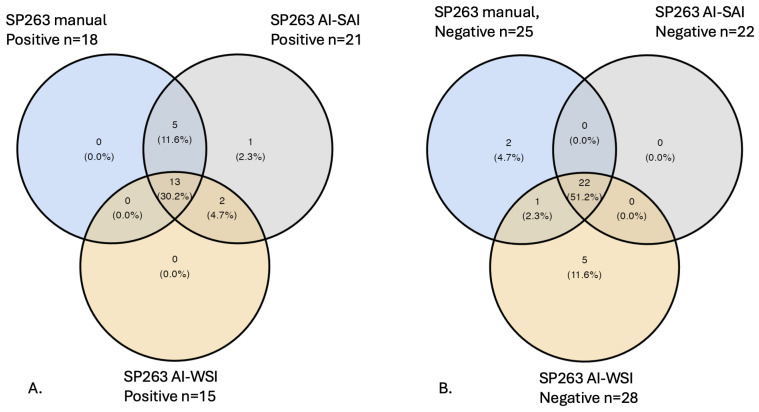
Venn diagrams showing the distribution of positive (**A**) and negative (**B**) PD-L1 cases between the three protocols: manual interpretation, AI-SAI, and AI-WSI protocols. PD-L1-positive cases (**A**) were distributed among the three protocols. Thus, there are cases common to all three protocols, cases common to only two of them, and cases common to only one protocol. The same procedure is also performed for PD-L1-negative cases (**B**). PD-L1: Programmed Death Ligand 1; AI-SAI: Artificial Intelligence protocol, Selected Area Interpretation; AI-WSI: Artificial Intelligence protocol, Whole Slide Imaging.

To analyze the concordance and discordance between the three interpretation methods, we constructed Venn diagrams (Figure 4A,B).

When applying the manual interpretation model (the gold standard), 18/43 cases were identified as positive; of these, 13 cases were concordantly positive with both the AI-SAI and AI-WSI protocols, and 5 cases were concordantly positive with the AI-SAI protocol only. Two cases that were positive with both the AI-SAI and AI-WSI protocols were scored as negative when assessed via manual interpretation. One case was positive exclusively when evaluated using the AI-SAI protocol (Figure 4A). With regard to the negative cases, 25/43 BCs were scored as PD-L1-negative when applying the manual interpretation model (the gold standard); of these, 22 cases were concordantly negative with both the AI-SAI and AI-WSI protocols, and 1 case was concordantly negative with the AI-WSI protocol only. Five cases were exclusively negative with the AI-WSI protocol (Figure 4B).

Pairwise comparison using kappa analysis on PD-L1 scoring with the three methods demonstrated good to very good agreement. The results indicated a high concordance between the AI-SAI scoring protocol and the manual evaluation, showing a 93% agreement level and a kappa value of 0.86 (very good) (*p* < 0.001). Comparing the AI-WSI protocol with the manual evaluation resulted in an 84% agreement level and a kappa value of 0.65 (good) (*p* < 0.001).

**Figure 5:**
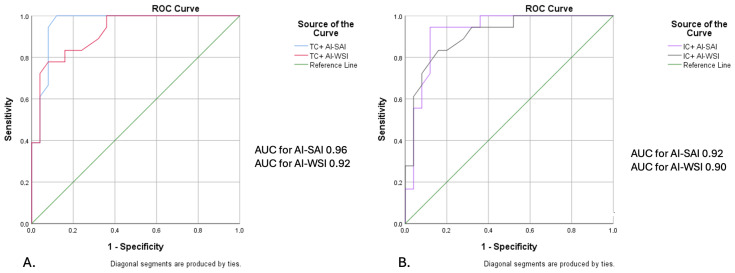
Comparative ROC curve analysis for assessing the diagnostic accuracy of the AI-SAI and AI-WSI protocols in relation to manual interpretation. ROC curve analysis was performed to assess the performance of AI-SAI and AI-WSI versus manual interpretation since their corresponding sensitivity and specificity values are insufficient. This procedure assessed the detection of TCs (**A**) and ICs (**B**) using both protocols with respect to manual interpretation, which was used as the gold standard. As revealed by the AUC values, the AI-SAI protocol exceeded the AI-WSI protocol in assessing both TCs and ICs. ROC: Receiver Operating Characteristic; AI-SAI: Artificial Intelligence protocol, Selected Area Interpretation; AI-WSI: Artificial Intelligence protocol, Whole Slide Imaging; TCs: tumor cells; ICs: immune cells.

Considering manual interpretation as the gold standard, the AI-SAI protocol exhibited 100% sensitivity, 88% specificity, 93% diagnostic accuracy, 85.7% positive predictive value, and 100% negative predictive value. In comparison to the gold standard (manual interpretation), the AI-WSI protocol demonstrated a sensitivity of 72%, a specificity of 92%, a diagnostic accuracy of 83.7%, a positive predictive value of 86.7%, and a negative predictive value of 82.1%. ROC curve analysis for TCs indicated an AUC of 0.96 for the AI-SAI protocol and 0.92 for the AI-WSI protocol. For ICs, the AUC values were recorded as 0.92 for AI-SAI and 0.90 for AI-WSI. Consequently, when comparing the two protocols, AI-SAI exhibited superior performance relative to AI-WSI, which followed closely (Figure 5A,B).

## 4. Discussion

### 4.1. Digital Pathology in Diagnostic Medicine

Digital slide imaging is becoming increasingly popular in pathology, with AI algorithms addressing traditional challenges in pathological assessments, such as the labor-intensiveness and subjectivity in PD-L1 scoring. Although AI algorithms hold considerable potential for enhancing PD-L1 assessment, existing research indicates that AI cannot yet fully replace human pathologists [18]. The outcomes of AI interpretation are still highly variable, ranging from unsatisfactory to promising and encouraging, depending on factors such as software and expertise in using AI tools. Nevertheless, the FDA has approved the use of WSI for primary diagnosis in surgical pathology and granted clearance for the use of an AI algorithm in the histopathological diagnosis of prostate cancer [19,20,21].

### 4.2. An Overview of the Results of the Current Study

Our study aimed to test the efficiency of AI-driven software suite interpretation for PD-L1 IHC assessment in BC cases and compare it to conventional manual scoring evaluation, which served as the gold standard. We chose the free open-source QuPath software as a candidate since it offers several important advantages: (1) it does not require special programming skills; (2) it is user-friendly; and (3) it has an Artificial Intelligence (AI) component that can easily be “taught” to assess PD-L1 IHC. Thus, we set up and standardized two protocols for automatic PD-L1 scoring assessment: a custom selection of representative areas (the AI-SAI protocol) and the inclusion of the whole slide image (the AI-WSI protocol). These two AI protocols were compared to the standard manual PD-L1 IHC interpretation provided by three experienced pathologists that had examined all the slides in consensus. Our results show good and very good test performances (in terms of sensitivity, specificity, diagnostic accuracy, and positive and negative predictive values) for both the AI-SAI and AI-WSI protocols, although they are inferior to manual interpretation. Nevertheless, AI-SAI exceeded AI-WSI in assessing both TCs and ICs, the latter being slightly behind.

### 4.3. Placing Our Findings Within the Existing Literature Context

Our findings confirm prior reports, with minor variations. In the assessment of TCs, we observed very good and good agreement levels for both the AI-SAI (0.86, *p* < 0.001) and AI-WSI (0.65, *p* < 0.001) protocols in comparison to manual interpretation. Similarly, Plass et al. [19] evaluated AI interpretation for PD-L1 staining in NSCLC patients using two commercially available software platforms. Their study reported lower kappa agreement results compared to ours (0.354 for uPath software, Roche, Basel, Switzerland and 0.672 for the PD-L1 Lung Cancer TME application—Visiopharm suite, Hoersholm, Denmark). They highlighted the necessity for further refinement of AI tools to achieve reliability comparable to evaluations conducted by human experts.

In an effort to explore the interpretation of PD-L1 staining in BC cases using AI, Rüschoff et al. [22] conducted a comprehensive multicenter international study. They compared the manual assessment of two PD-L1 IHC clones, 22C3 and 28-8, each with different interpretation systems, to a highly trained in-house AI system for PD-L1 scoring in BCs. The authors reported lower agreement rates than those observed in our study, with kappa values of 0.42 (95%CI 0.26–0.55) for the 28-8 clone and 0.64 (95%CI 0.49–0.77) for the 22C3 clone.

We obtained lower agreement rates compared to the results published by Lee et al. [23]. In their multi-institutional study, they assessed the AI interpretation of PD-L1 staining with the 22C3 clone on BC cases. They used a custom AI system (Lunit Scope PD-L1, Lunit Inc., Seoul, Republic of Korea) that was trained on 400 WSI images and obtained an agreement of 89.5% in WSI using CPS (combined positive score). This was further improved by reviewing discordant cases, reaching a total agreement rate of 92.4%.

Spearman tests were conducted to explore correlations for TC and IC assessments between the manual interpretation, AI-SAI, and AI-WSI protocols. Very good correlations were found in evaluating TCs between the three methods (r > 0.8), and moderate to good correlations were found in evaluating ICs (0.55 < r < 0.75). These results align with the findings reported by Taylor et al. [11], who tested the efficiency of the Optra system (OptraSCAN Inc., San Jose, CA, USA) for both slide scanning and PD-L1 interpretation in a series of NSCLC cases using the PD-L1 22C3 clone. Their study found higher levels of agreement for the assessment of TCs compared to ICs.

In their study, Naso et al. [10] investigated the efficiency of the SAI protocol in assessing PD-L1 expression (22C3 clone) in a series of NSCLCs by comparing it to manual PD-L1 interpretation and evaluating the percentage of positive TCs in selected image patches from digital slides. The authors reported kappa values that were similar to those in our study (0.8 when a 1% threshold was applied to TCs and 0.78 when the 50% threshold was applied versus 0.86 in our study).

### 4.4. What About the ICs?

We observed high agreement rates for evaluating TCs when using both the AI-SAI and AI-WSI protocols. Conversely, this did not apply to ICs, which were more complex to assess. In line with existing literature data, our findings highlight the challenges of evaluating ICs with AI systems, as they can be affected by confounding factors such as prior transurethral resection lesions, resulting in ulceration, necrosis, and infiltration by giant multinucleated cells, which may introduce bias into the AI model [23]. These factors could explain the general difficulty of AI systems in interpreting staining on ICs [10].

### 4.5. Study’s Strengths and Weaknesses

Our study is subject to several limitations. The sample size is one important limitation. Nevertheless, we addressed this issue and overcame it by incorporating multiple detection areas for AI training from each specific case. Additionally, all samples were retrospectively selected from a single institution. Future research should encompass larger multicenter prospective studies to evaluate various PD-L1 clones and assess whether different processing protocols influence test performance. Moreover, improved refinement of this community-driven, open-source software could facilitate its integration into routine pathological practice.

Another limitation of our study is the lack of a formal assessment of interobserver variability. However, this was addressed through a consensus evaluation of the study slides by the three pathologists using a three-headed microscope.

Our study is distinguished by its highly innovative research concept and by the results we obtained. PD-L1 IHC automatic scoring using AI algorithms is becoming increasingly popular in pathology as it may offer a potential way to achieve high-throughput PD-L1 IHC scoring. Our study has demonstrated that automated PD-L1 scoring for BC cases has an overall lower accuracy when compared to manual slide interpretation, with a tendency to overestimate scores in specific situations. Nonetheless, QuPath software can be employed in clinical practice after thorough calibration of the AI classifier and may provide help in PD-L1 IHC testing workflows.

## 5. Conclusions

Automated AI-driven PD-L1 IHC staining interpretation is a feasible, useful tool that holds promise for application in daily clinical practice. In our study, we have demonstrated that AI systems show very good agreement rates with manual interpretation, especially for BC cases that are rich in PD-L1-positive TCs. For cases that are rich in PD-L1-positive ICs or with borderline positive ratios, supervision by an experienced pathologist is mandatory.

Although the AI-SAI protocol was the closest to manual PD-L1 interpretation, there is a further necessity for pathologists to support the QuPath software PD-L1 interpretation for an accurate diagnosis. Nevertheless, in the future, the use of AI algorithms could become a valuable tool for pathologists when scoring PD-L1.

## Figures and Tables

**Table 1:** Clinical and pathological characteristics of the study cases.

Characteristic	Total (*n* = 43)
Demographic data	
Age (mean ± SD)	63.5 ± 7.4
Male (*n*, %)	36 (84%)
Female (*n*, %)	7 (16%)
Pathological features (*n*, %)	
Histology	
UC conventional	23 (53.5%)
UC variants	
Poorly differentiated	6 (14%)
Sarcomatoid	1 (2.3%)
Squamous	4 (9.3%)
Micropapillary	3 (7%)
Plasmacytoid	3 (7%)
Glandular	1 (2.3%)
Mixed (glandular and squamous)	2 (4.7%)
TNM staging	
Primary tumor, pT (*n*, %)	
T2	13 (30.2%)
T3	20 (46.5%)
T4	10 (23.3%)
Lymph node involvement	30/43 (69.7%)
Distant metastasis	5 (11.6%)

UC: urothelial carcinoma; SD: standard deviation; TNM: Tumor Lymph Node Metastasis classification.

**Table 2:** Comparative analysis of PD-L1 staining (tumor cells, immune cells, and PD-L1 status) between the three assessment methods: manual versus AI-driven protocols (AI-SAI and AI-WSI).

Factors	PD-L1 Manual	PD-L1 AI-SAI	PD-L1 AI-WSI	*p* Value
TCs (*n*, %)				*p* = 0.008 *
%TCs positive (≥25%)	17/39.5%	19/44.18%	14/32.5%	
%TCs negative (<25%)	26/60.4%	24/55.8%	29/67.4%	
ICs (*n*, %)				
%ICs positive (≥25%)	3/6.9%	17/39.5%	15/34.8	*p* < 0.001 *
%ICs negative (<25%)	40/93.1%	26/60.4%	28/65.1	
PD-L1 status Combined TCs%/ICs% (*n*, %)				
Positive	18/42%	21/48.8%	15/34.8	*p* = 0.034 *
Negative	25/58%	22/51.2%	28/65.1	

AI-SAI: Artificial Intelligence protocol, Selected Area Interpretation; AI-WSI: Artificial Intelligence protocol, Whole Slide Imaging; TC: tumor cell; IC: immune cell; PD-L1: Programmed Death Ligand 1. * Statistical comparison was performed using the Friedman test.

## Data Availability

The data presented in this study are available on request from the corresponding author due to privacy and ethical reasons.

## Abbreviations

The following abbreviations are used in this manuscript:

AI	Artificial Intelligence
AI-SAI	Artificial Intelligence protocol, Selected Area Interpretation
AI-WSI	Artificial Intelligence protocol, Whole Slide Imaging
AJCC/UICC	American Joint Committee on Cancer/Union for International Cancer Control
AUC	Area Under the Curve
BC	Bladder cancer
CPS	Combined positive score
DAB	Diaminobenzidine
EDTA	Ethylenediaminetetraacetic acid
EMA	European Medicines Agency
FDA	Food and Drug Administration
FFPE	Formalin-fixed and paraffin-embedded
HE	Hematoxylin and eosin
ICs	Tumor-infiltrating immune cells
IHC	Immunohistochemistry
NSCLC	Non-small cell lung cancer
PD-1	Programmed Death 1
PD-L1	Programmed Death Ligand 1
ROC	Receiver Operating Characteristic
TCs	Tumor cells
TNM	Tumor Lymph Node Metastasis
UC	Urothelial carcinoma
WHO	World Health Organization
