# Correction: Nechifor-Boilă et al. Artificial Intelligence (AI) for Programmed Death Ligand-1 (PD-L1) Immunohistochemical Assessment in Urothelial Carcinomas: “Teaching” Cell Differentiation to AI Systems. *Life* 2025, *15*, 839

**DOI:** 10.3390/life15081297

**Published:** 2025-08-15

**Authors:** Ioan Alin Nechifor-Boilă, Adela Nechifor-Boilă, Andrada Loghin, Carmen Mihaela Mihu, Carmen Stanca Melincovici, Mădălin Mihai Onofrei, Călin Bogdan Chibelean, Orsolya Martha, Angela Borda

**Affiliations:** 1 Department of Anatomy and Embryology, George Emil Palade University of Medicine, Pharmacy, Science and Technology of Targu-Mures, 38th Gh. Marinescu Street, 540142 Targu-Mures, Romania; ioan.nechifor-boila@umfst.ro; 2 Department of Urology, Targu-Mures County Hospital, 1st Gh. Marinescu Street, 540103 Targu-Mures, Romania; calin.chibelean@umfst.ro (C.B.C.); orsolya.martha@umfst.ro (O.M.); 3 Department of Histology, Center for Advanced Medical and Pharmaceutical Research, George Emil Palade University of Medicine, Pharmacy, Science and Technology of Targu-Mures, 38th Gh. Marinescu Street, 540142 Targu-Mures, Romania; andrada.loghin@umfst.ro (A.L.); angela.borda@umfst.ro (A.B.); 4 Department of Pathology, Targu-Mures County Hospital, 28 1st December 1918 Boulevard, 540011 Targu-Mures, Romania; 5 Discipline of Histology, Department of Morpho-Functional Sciences, Iuliu Hatieganu University of Medicine and Pharmacy, 400349 Cluj-Napoca, Romania; carmenmihu@umfcluj.ro (C.M.M.); carmen.melincovici@umfcluj.ro (C.S.M.); madalin.onofrei@umfcluj.ro (M.M.O.); 6 Department of Urology, George Emil Palade University of Medicine, Pharmacy, Science and Technology of Targu-Mures, 38th Gh. Marinescu Street, 540142 Targu-Mures, Romania; 7 Department of Pathology, Targu-Mures Emergency County Hospital, 50th Gh. Marinescu Street, 540136 Targu-Mures, Romania

## Additional Affiliation(s)

In the published publication [1], there was an error regarding the affiliations for Călin Bogdan Chibelean and Orsolya Martha. In addition to affiliation 2, the updated affiliation(s) should include: Department of Urology, George Emil Palade University of Medicine, Pharmacy, Science and Technology of Targu-Mures, 38th Gh. Marinescu Street, 540142 Targu-Mures, Romania (affiliation no. 6). The authors state that the scientific conclusions are unaffected. This correction was approved by the Academic Editor. The original publication has also been updated.
